# Structure-activity assessment of flavonoids as modulators of copper transport

**DOI:** 10.3389/fchem.2022.972198

**Published:** 2022-08-23

**Authors:** Vanessa J. Lee, Marie C. Heffern

**Affiliations:** Department of Chemistry, University of California, Davis, Davis, CA, United States

**Keywords:** flavonoids, copper trafficking, antioxidant activity, ionophore, chelator

## Abstract

Flavonoids are polyphenolic small molecules that are abundant in plant products and are largely recognized for their beneficial health effects. Possessing both antioxidant and prooxidant properties, flavonoids have complex behavior in biological systems. The presented work investigates the intersection between the biological activity of flavonoids and their interactions with copper ions. Copper is required for the proper functioning of biological systems. As such, dysregulation of copper is associated with metabolic disease states such as diabetes and Wilson’s disease. There is evidence that flavonoids bind copper ions, but the biological implications of their interactions remain unclear. Better understanding these interactions will provide insight into the mechanisms of flavonoids’ biological behavior and can inform potential therapeutic targets. We employed a variety of spectroscopic techniques to study flavonoid-Cu(II) binding and radical scavenging activities. We identified structural moieties important in flavonoid-copper interactions which relate to ring substitution but not the traditional structural subclassifications. The biological effects of the investigated flavonoids specifically on copper trafficking were assessed in knockout yeast models as well as in human hepatocytes. The copper modulating abilities of strong copper-binding flavonoids were largely influenced by the relative hydrophobicities. Combined, these spectroscopic and biological data help elucidate the intricate nature of flavonoids in affecting copper transport and open avenues to inform dietary recommendations and therapeutic development.

## 1 Introduction

**FIGURE 1:**
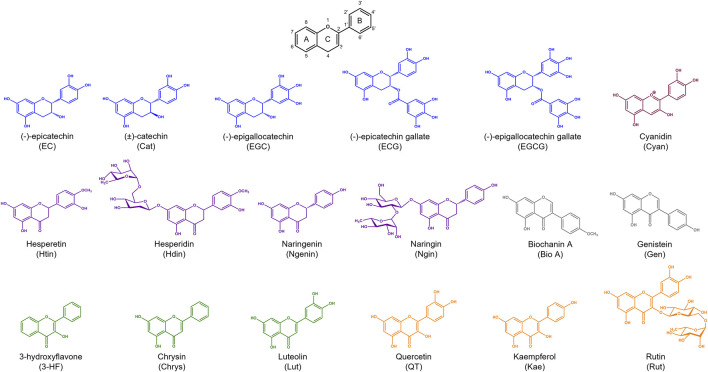
The core structure shared by flavonoids is composed of two benzene rings (rings A and B) connected by a heterocyclic pyran ring (ring C). Flavonoid subclasses include flavonols (blue), flavanones (purple), flavones (green), flavonols (orange), anthocyanins (maroon), and isoflavones (grey).

Flavonoids are a class of small phenolic secondary plant metabolites. Ubiquitous in the plant kingdom, flavonoids are recognized as micronutrients that are biologically active in mammalian systems (Datta et al., 2004). Flavonoids are widely studied for their health benefits which include anti-inflammatory and anti-cancer properties (Dai et al., 2017). However, the mechanisms of their biological activity remain unclear due to flavonoids’ complex activity as both anti- and pro-oxidants. Flavonoids share a core structure containing two benzene rings connected by a heterocyclic pyran ring (Figure 1). Derivatization of this core structure differentiates flavonoids into subclasses based on oxidation and substitution of the heterocyclic C-ring (Table 1). Modest structural differences correspond to changes in biological effects with variations in hydroxyl group positions relating to differing antioxidant activity (Rice-Evans et al., 1996). Often, flavonoids are studied by subclasses with the assumption that molecules within subclasses behave similarly.

**TABLE 1:** Structures and subclasses of flavonoids.

Subclass	Structure	Flavonoids	Substitutions
Flavan-3-ols	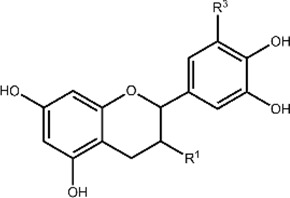	Epicatechin (EC)	R^1^ = OH, R^2^ = H
		Catechin (Cat)	R^1^ = OH, R^2^ = H
		Epigallocatechin (EGC)	R^1^ = OH, R^2^ = OH
		Epicatechin gallate (ECG)	R^1^ = gallate, R^2^ = H
		Epigallocatechin gallate (EGCG)	R^1^ = gallate, R^2^ = OH
Flavanones	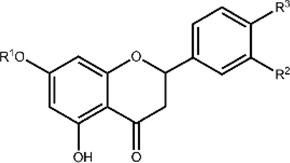	Hesperetin (Htin)	R^1^ = H, R^2^ = OH, R^3^ = OCH_3_
		Hesperidin (Hdin)	R^1^ = glucoside, R^2^ = OH, R^3^ = OCH_3_
		Naringenin (Ngenin)	R^1^ = H, R^2^ = H, R^3^ = OH
		Naringin (Ngin)	R^1^ = glycoside, R^2^ = H, R^3^ = OH
Flavones	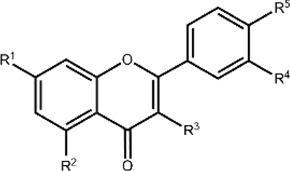	Luteolin (Lut)	R^1^ = OH, R^2^ = OH, R^3^ = H, R^4^ = OH, R^5^ = OH
		Chrysin (Chrys)	R^1^ = OH, R^2^ = OH, R^3^ = H, R^4^ = H, R^5^ = H
		3-hydroxyflavone (3-HF)	R^1^ = H, R^2^ = H, R^3^ = OH, R^4^ = H, R^5^ = H
Flavonols	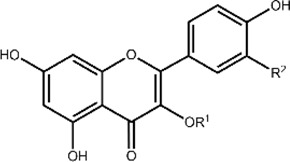	Quercetin (QT)	R^1^ = H, R^2^ = OH
		Kaempferol (Kae)	R^1^ = H, R^2^ = H
		Rutin (Rut)	R^1^ = rutinoside, R^2^ = OH
Anthocyanins	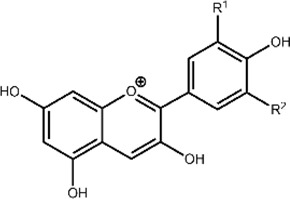	Cyanidin (Cyan)	R^1^ = H, R^2^ = OH
Isoflavones	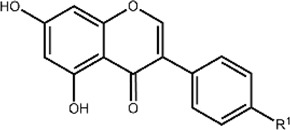	Biochanin A (Bio A)	R^1^ = OCH_3_
		Genistein (Gen)	R^1^ = OH

Interactions of flavonoids with metal ions are a source of both anti- and pro-oxidant activity (Perron et al., 2011). Flavonoids have been shown to perform Fenton-type reactions in the presence of Fe(III) and Cu(II) ions (Fang et al., 2020). Furthermore, coordination to metal ions affects the oxidative activity of flavonoids. There is evidence that flavonoids interact with metal ions including Fe(III), Zn(II), and Cu(II) with varying reports on the potential biological effects (Weber 1988; Escandar and Sala 1991; Fernandez et al., 2002; Ren et al., 2008; Pekal et al., 2011; Říha et al., 2014; Anderson, Larson, and Berreau 2016; Cherrak et al., 2016).

While copper is an essential trace metal micronutrient, its dysregulation and mislocalization is correlated with a host of pathologies including Wilson’s disease, neurodegeneration, and some cancers (Baldari et al., 2020). The causal relationship between these pathologies and copper dysregulation is still undetermined, but redirecting copper trafficking, either by removal or altering the localization of the metal ion, is emerging as a viable therapeutic strategy (Hunsaker and Franz 2019). For instance, copper chelation therapies have shown encouraging effects in reducing morbidity in Wilson’s disease patients and restricting tumor angiogenesis (Baldari et al., 2020). By in large, the focus on copper-modulating agents have been on synthetic chelators and ionophores, with less attention paid to natural products such as flavonoids. Investigating the copper-modulating properties of flavonoids may not only facilitate rational design but may also inform on nutrition-based effects of such plant-derived products. As flavonoids display metal chelating properties, their health benefits may in part be due to their ability to affect copper trafficking (Říha et al., 2014).

Indeed, some flavonoids reportedly protect against symptoms of copper-dysregulation associated pathologies (Cremonini et al., 2018), but the structure/function relationship between how their chelating abilities relate to their potential therapeutic actions in these scenarios requires further elucidation. To this end, this work sought to elucidate the relationship between flavonoid-copper interactions and their ability to affect copper trafficking in cell-based models. A library of 18 flavonoids was compiled composed of flavonoids that are bioavailable through food consumption and recognized for their health benefits. The Cu(II)-binding properties of the 18 flavonoids spanning the subclasses (Table 1) were directly compared spectroscopically, and their impact on Cu(II)-associated redox activity was assessed with *in vitro* assays. Flavonoids with appreciable Cu(II)-binding abilities were assessed for their ability to alter copper trafficking in cell-based eukaryotic models. Taken together, this study offers a deeper understanding of how specific structural features of flavonoids may relate to their potential therapeutic activity in copper-associated disorders.

## 2 Materials and methods

All chemicals were used as purchased without further purification. (-)-Epicatechin (EC), (-)-epicatechin gallate (ECG), quercetin (QT), 3-hydroxyflavone (3-HF), hesperetin, ACES, tricine, and bathocuproine disulfonic acid disodium salt (BCS) were obtained from Sigma-Aldrich (St. Louis, MO). Catechin (Cat), luteolin (Lut), (-)-epigallocatechin (EGC), and biochanin A were purchased from VWR (Radnor, PA). (-)-Epigallocatechin gallate (EGCG), chrysin, naringenin, naringin, hesperidin, rutin, kaempferol, cyanidin, genistein, hematoxylin, coumarin-3-carboxylic acid (CCA), ascorbic acid, ethylenediaminetetraacetic acid (EDTA), tris(hydroxymethyl-d_3_)amino-d_2_-methane (deuterated Tris, Tris-d_11_), MOPS, BisTris, and all solvents were obtained from Fisher Scientific (Waltham, MA).

### 2.1 Determination of binding ratio and binding affinity

All UV-Vis studies were performed on a Shimadzu UV-1900i at 30°C or 37°C using quartz cuvettes (Starna) with a pathlength of 1 cm. Milli-Q water was the reference for all studies, and the spectrum of the buffer was subtracted, and the spectra were normalized after data collection.

50 μM solutions of flavonoids were prepared in 50 mM buffer (pH 7.4). CuSO_4_, dissolved in Milli-Q water, was titrated into the solution in ratios ranging from 10–250 μM.

### 2.2 Identification of binding location

#### 2.2.1 ^1^H Studies

Spectra were recorded on a Bruker 400 MHz NMR spectrometer with Topspin 3.2 running IconNMR 50 mM solutions of flavonoids were prepared in DMSO-d_6_ and 12.5 mM solutions of CuSO_4_ were prepared in MeOD. Samples were prepared of flavonoids alone at a final concentration of 12.5 mM flavonoid and with 0.25 equivalents of CuSO_4_ dissolved in 50:50 DMSO-d_6_:MeOD.

### 2.3 DPPH radical scavenging of flavonoids

The radical scavenging abilities of flavonoids were studied using the DPPH^•^ assay as previously described (Xie and Schaich 2014; Zhao et al., 2020). In short, 50 μM 2,2-diphenyl-1-picrylhydrazyl free radical (DPPH) in ethanol was added to preincubated solutions of 50 μM flavonoid, or ascorbic acid as the positive control, with and without 50 μM CuSO_4_ or ZnCl_2_ in a 96-well plate. Time dependent absorbance measurements were recorded at 515 nm over the course of 60 min using a Spectramax i3x microplate reader (Molecular Devices, San Jose, CA). The radical scavenging ability was calculated using the following equation:

$$\mathrm{DPPH}\;\mathrm{scavenging}\%=\left[1-\left(A_{\mathrm{sample}}-A_{\mathrm{flav}}\right)/A_{\mathrm{DPPH}}\right]\times 100\%$$ (1)

where A_sample_ is the absorbance of the flavonoid and DPPH, A_flav_ is the absorbance of the flavonoid alone, and A_DPPH_ is the absorbance of DPPH alone at the end of the 1-h measurement.

### 2.4 Measurement of ^•^OH in solution

The amount of ^•^OH in solution was measured as previously described (Hu et al., 2016). A solution of 2.5 mM CCA and 500 μM ascorbic acid was prepared in 10 mM phosphate buffer, pH 7.4. 50 μl of the CCA/ascorbic acid solution was added to 200 μl of pre-incubated flavonoid/CuSO_4_ solutions at the described concentrations. Time-dependent fluorescence intensity measurements were recorded with excitation at 388 nm and emission at 450 nm over the course of 90 min using a Spectramax i3x microplate reader (Molecular Devices, San Jose, CA).

### 2.5 Assessment of copper transport in yeast

*Saccharomyces cerevisiae* strains used in this study are listed in Supplementary Table S2. Yeast cells were cultured in YPD (1% yeast extract, 2% peptone, 2% glucose) and YPGE (1% yeast extract, 2% peptone, 3% glycerol, 2% ethanol) media. Growth in liquid YPD media was monitored at 600 nm. For qualitative observations, spot plate assays were performed on YPD and YPGE plates supplemented with the indicated flavonoids and copper. 10-fold serial dilutions of overnight YPD cultures were spotted on YPGE agar plates supplemented with 25 μM flavonoids or 5 μM CuSO4 and incubated at 37°C for the indicated time.

### 2.6 HepG2 cell culture and stimulations

HepG2 cells were maintained in 4.5 g/L DMEM media supplemented with 10% fetal bovine serum, 1% sodium pyruvate, 1% glutamine, 1% Penicillin-Strepomycin. Cells were incubated at 37°C with 5% CO_2_ in complete media until 70% confluence. HepG2 cells were plated at 300,000 cells/well in 6-well plates and allowed to grow for 24 h. Stimulations of 20 μM flavonoid with and without 50 μM CuSO4 were added to the wells and incubated for 24 h.

### 2.7 Cell viability

Cell viability was measured using the MTS assay (Promega, Madison, WI). HepG2 cells were plated at 10,000 cells/well in a 96-well plate and allowed to grow for 24 h. Wells were stimulated with 20 μM flavonoid with and without 50 μM CuSO4. After 24 h, media was removed, and cells were washed with DPBS to remove excess flavonoid. 20 μl of MTS reagent was added to each well and incubated for 1 h. Absorbance at 490 nm was measured.

### 2.8 Intracellular copper measurements

After stimulations, HepG2 cells were washed with cold 50 mM EDTA in HEPES buffer to remove any copper on the cell surface followed by washes with HEPES buffer. Cells were resuspended in 50 μl HEPES buffer and added to 250 ml 70% nitric acid. Samples were boiled for 1 h at 95°C then allowed to sit at room temperature for 24 h. Samples were diluted to 5 ml with nanopure water. Metal analysis was performed using a Perking Elmer 5300 DV optical emission ICP with auto sampler. Measured copper levels were normalized to the control sample.

### 2.9 Western blot analysis

After stimulations, HepG2 cells were washed with DPBS and lysed with 60 μl RIPA buffer. Whole cell lysates were centrifuged at 15,000 rpm at 4°C for 1 h. The supernatant was collected and stored at −20°C. Protein quantification was determined using the BCA assay. 20 μg of protein sample was loaded into 15-well SDS-PAGE gels and transferred to polyvinylidene difluoride (PVDF) membranes. PVDF membranes were incubated at 4°C overnight with primary antibodies. Primary antibodies used were anti-CCS (1:2000, sc-55561, Santa Cruz Biotechnology, anti-mouse), and anti-α-tubulin (1:10,000, MA1-80017, ThermoFisher, anti-rat). Primary antibodies were removed, and the membranes were washed with tris buffered saline with Tween (TBST). Membranes were then incubated in secondary antibodies at room temperature for 1 h. Secondary antibodies used were anti-mouse IgG AlexaFluor800 (A32789, 1:5000 Invitrogen) and anti-rat IgG (A21428, 1:5000 Invitrogen), AlexaFluor647, and a horseradish peroxidase-conjugated secondary antibody. Blot images were recorded on a BioRad ChemiDoc imaging system and processed in ImageLab.

## 3 Results and discussion

### 3.1 Spectroscopic characterization of flavonoid-copper binding interactions in solution

**FIGURE 2:**
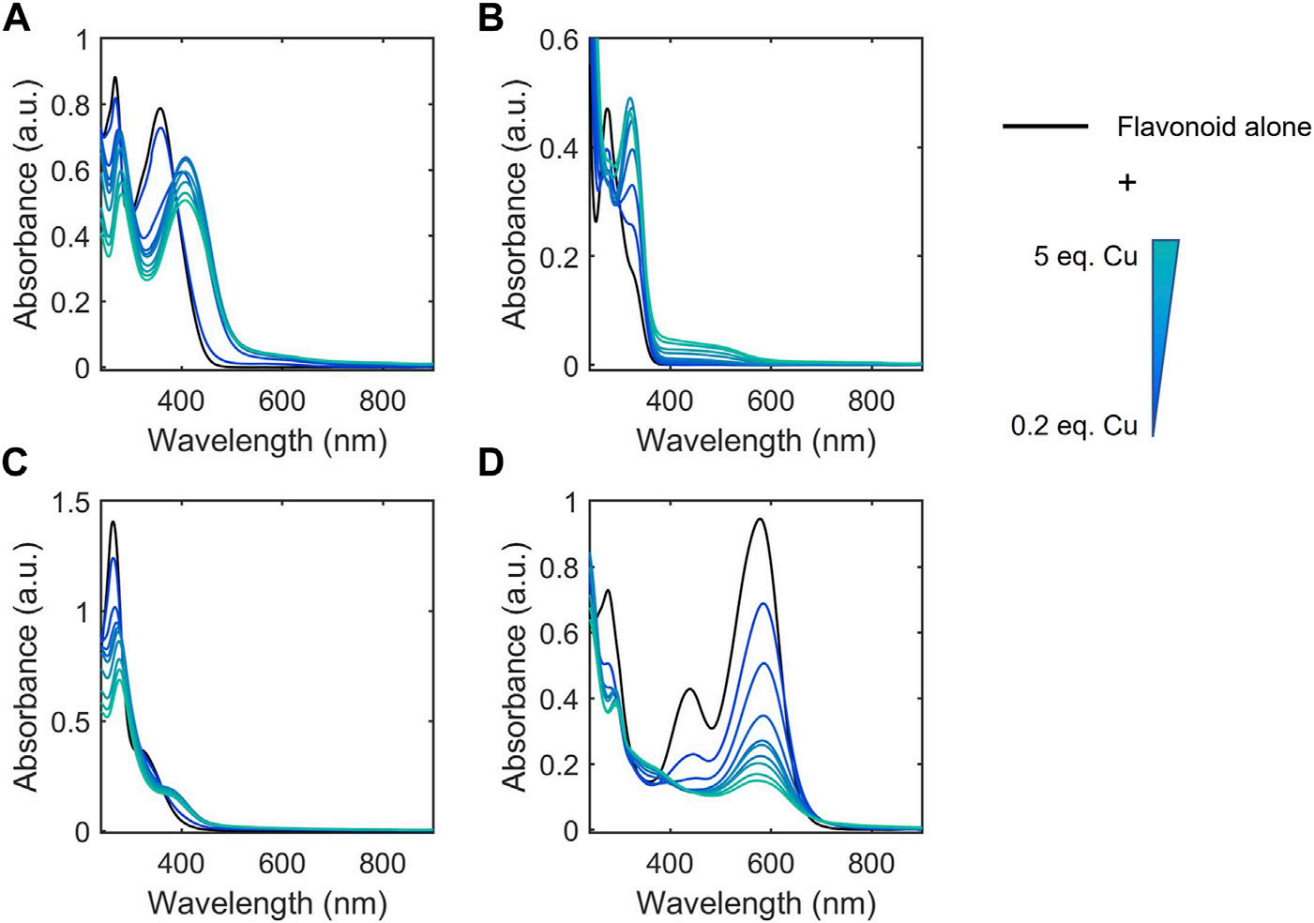
Electronic absorption spectra of representative flavonoids **(A)** luteolin, **(B)** EGCG, **(C)** biochanin A, and **(D)** cyanidin (black) in 50 mM MOPS buffer (pH 7.4) titrated with CuSO_4_. Flavonoids were prepared at 50 µM and CuSO_4_ was titrated into the solution from 0.2 to 5 molar equivalences. Flavonoids typically have two distinct absorption bands: a strong absorbance between 270 and 295 nm (Band II) and a weak Band I absorbance that appears as a shoulder at slightly longer wavelengths. Changes in the intensities and shifts in the lambda max wavelengths of these bands, indicate interactions of Cu(II) with the flavonoids. Electronic absorption spectra of the remaining flavonoids tested are shown in Supplementary Figure S1.

A breadth of techniques have been applied to probe aspects of flavonoid-metal interactions including computational methods, electrochemical studies, and various spectroscopies (Korotkova et al., 2012; Samsonowicz and Regulska 2017; Xu et al., 2020). However, reports have used varying experimental conditions including solvents, buffers, and pH conditions, yielding contradicting parameters for flavonoid-metal interactions (Samsonowicz and Regulska 2017). Thus, to allow connections to be made between structural features and biological function, we applied a universal approach that would allow us to comparatively study our selected library of flavonoids across the subclasses. The conjugated structure of flavonoids lends itself to analysis by absorption spectroscopy. There are two major peaks in UV-Vis spectra for flavonoids. Band I corresponds to absorbance by the B ring while Band II appears at lower wavelengths due to absorbance by the A ring (Figure 2A). (Kumar and Pandey 2013) Flavanols and flavanones do not have conjugation between the A and B rings. This lack of conjugation is reflected in the presence of a strong absorbance between 270 and 295 nm (Band II) and a weak Band I absorbance that appears as a shoulder at slightly longer wavelengths (Figure 2B). (Rice-Evans et al., 1996)

**FIGURE 3:**
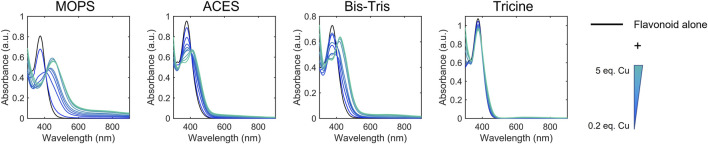
Representative electronic absorbance spectra of QT and CuSO_4_ in various 50 mM buffers (pH 7.4) for determining binding affinity ranges. CuSO_4_ was titrated into a 50 μM solution of QT from 0.2 to 5 equivalences. Spectral changes correspond to binding between QT and Cu(II) which indicates that QT has a stronger binding affinity than the corresponding buffer. Conversely, the lack of spectral changes upon Cu(II) addition in tricine buffer indicates that the binding affinity between tricine and Cu(II) is stronger than that of QT and Cu(II). These experiments allow us to estimate the binding affinity of QT and Cu(II) to be between that of BisTris-Cu(II) (5.3 μM) and tricine-Cu(II) (50 nM). Electronic absorption spectra of the remaining flavonoids tested are shown in Supplementary Figure S1.

Cu(II) binding near or at sites of conjugation yields spectral changes to these bands, which indicate metal-flavonoid interactions. We used these spectral changes to monitor metal binding in the presence of Cu(II)-binding buffers to categorize the flavonoids based on approximate apparent binding affinity ranges. Flavonoids were classified into Cu(II) binding affinities based on their ability to compete for Cu(II) binding with 50 mM MOPS (K_d_ = 100 μM), ACES (K_d_ = 17.4 μM), BisTris (K_d_ = 5.3 μM), and Tricine (K_d_ = 50 nM) (Figure 3; Table 2). (Kandegedara and Rorabacher 1999; Atp 1979; Azab and El-Nady 1994; Azab et al., 2001; Fernandez et al., 2002) Spectra were recorded for the flavonoids alone in each of the buffers as well as after Cu(II) addition. Changes in the spectra upon Cu(II) addition indicate that the flavonoids have a greater affinity for Cu(II) than the buffer and therefore interact in solution. If the buffer has a greater binding affinity for Cu(II) than the flavonoid, no spectral changes are observed. All flavonoids tested showed binding in 50 mM MOPS, indicating K_d_ < 100 µM. While binding affinity ranges showed some correlation to flavonoid subclasses, some exceptions arise based on the type of functional group and position. The flavanones and isoflavones were all relatively weak binders with K_d_ >17.4 μM. While flavanols with -OH modifications at the C-3 position also showed relatively weak binding affinities with K_d_ >17.4 μM, the presence of a gallate modification at the C-3 position in epicatechin gallate (ECG) and epigallocatechin gallate (EGCG) increases the K_d_ of the flavanols to 5.3 μM–50 nM. This indicates that a gallate moiety on the C-ring can endow stronger Cu(II)-binding ability to this subclass, while, surprisingly, a catechol on the B-ring does not. Interestingly, the anthocyanin, cyanidin, which differs from epigallocatechin (EGC) by the introduction of conjugation between the A and C rings, shows a strong binding affinity, suggesting that such conjugation may contribute to the electron donating abilities of the flavonoid. Among the flavones, luteolin and 3-hydroxyflavone (3-HF) were strong binders with K_d_ between 5.3 μM and 50 nM whereas chrysin showed weak binding (K_d_ > 17.4 μM). Luteolin and chrysin only differ by the presence of a catechol moiety on the B-ring of the former, suggesting that Cu(II)-binding to luteolin likely occurs at this site. While the catechol moiety is absent in 3-HF, this flavone differs from chrysin by the presence of an -OH group in the C-3 position of the C-ring, suggesting that this functional group, likely in cooperation with the ketone at the C-4 position, coordinates to Cu(II). Of the flavonols, both quercetin (QT) and kaempferol are strong binders with K_d_ in the 5.3 μM–50 nM range. However, the C-3 O-rutinoside analogue of QT, rutin, showed reduced binding affinity (K_d_ in the 17.4–5.3 µM range), suggesting that this sugar modification disrupts Cu(II)-binding. The determined binding affinity ranges of QT and rutin support previously modeled binding affinities (Zhang et al., 2018).

**TABLE 2:** Binding affinity ranges of flavonoids with Cu(II).

Binding affinity ranges			
100 μM	17.4 μM	5.3 μM	50 nM
EC, cat, EGC, hesperetin, Hesperidin, naringenin, naringin, chrysin, biochanin A, genistein	Rutin	ECG, EGCG, 3-HF, Luteolin, QT, Kaempferol, Cyanidin	

**FIGURE 4:**
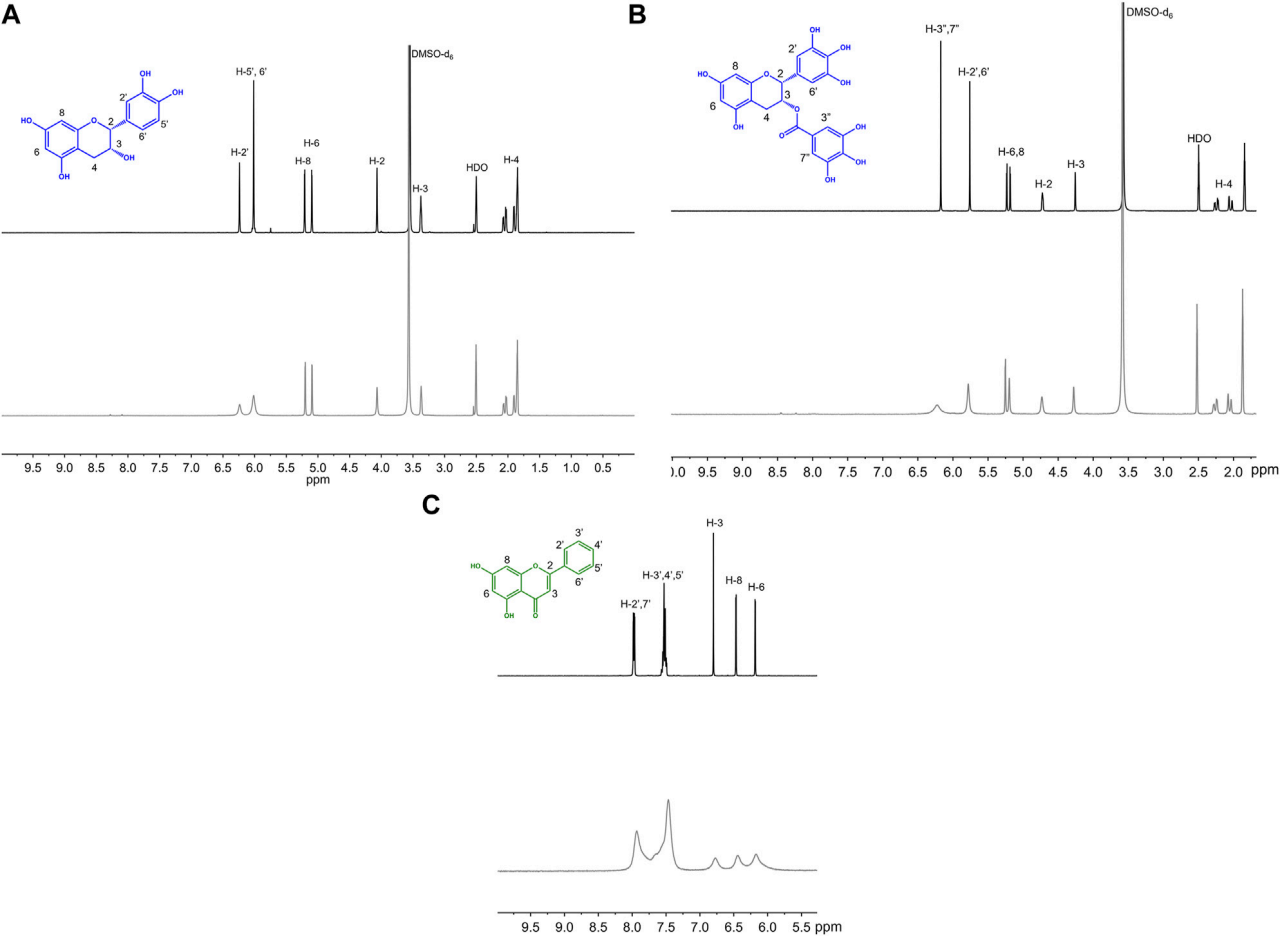
Representative ^1^H NMR spectra of 50 mM **(A)** EC, **(B)** EGCG, **(C)** chrysin with (gray) and without (black) 0.25 equivalents of CuSO_4_ in 50:50 DMSO-d_6_:MeOD.

To help further understand the contributions of specific structural features, we used the changes in absorption spectra to identify the location of Cu(II) interaction. Cu(II) interactions with the flavanols and flavanones increase the Band II absorbance, and flavanol-Cu(II) interactions also produce a bathochromic shift in Band I absorbance. These spectral shifts are attributed to changes in the conjugation on the B-ring where Cu(II) is hypothesized to interact. Flavonols and flavones likely interact with Cu(II) at the C-ring carbonyl which is supported by a bathochromic shift in Band I upon introduction of the metal ion in solution. Similar to the flavanones, the isoflavones show a large Band II absorbance and a smaller Band I absorbance that appears as a shoulder (Figure 2C). Cu(II) interactions cause a bathochromic shift in Band I absorbance likely due to interactions with the hydroxy group on the A-ring. Finally, the spectrum of cyanidin likewise shows a bathochromic shift in Band I upon addition of Cu(II) as well as significant decreases in absorbance (Figure 2D). These interaction locations were confirmed by ^1^H-NMR: as Cu(II) is paramagnetic, coordination will cause faster relaxation of nuclei within electronic proximity, resulting in observed broadening in the spectra. This property can be exploited to confirm the site of interaction on the flavonoid molecules. The addition of 0.25 equivalents of Cu(II) to epicatechin (EC) results in broadening of the downfield peaks which correspond to the B-ring protons (Figure 4A). (Davis et al., 1996) The localized broadening confirms the interaction of Cu(II) ions at the diol on the B-ring. The spectra of EGCG also present localized broadening around the most downfield protons (Figure 4B). The most downfield peak corresponds to the protons on the gallate group indicating that rather than interacting at the B-ring, Cu(II) ions interact at the gallate moiety (Delius et al., 2017). This confirms that it is indeed the interaction of Cu(II) with the gallate group that contributes to the stronger binding observed in the binding affinity estimation. It does appear that there is some interaction with the hydroxy groups on the B-ring evidenced by the large decrease in intensity of the peak at 5.8 ppm corresponding to the B-ring protons. Study of the flavanols is made easier by a saturated C-ring which results in no conjugation between the A- and B-rings and therefore more localized broadening. When conjugation is present throughout the molecule, introduction of paramagnetic Cu(II) to the flavonoid solutions results in universal broadening in the ^1^H-NMR spectra. Interpretation of spectra is more difficult with broadening across all peaks, but changes in intensity of peaks provide insight into the location of interaction. The spectrum of chrysin and Cu(II) shows broadening throughout the spectrum but has a significantly larger decrease in peak intensity of the peaks corresponding to the A- and C-ring protons (Figure 4C). (Ansari 2008) This confirms the expected interaction of Cu(II) with the 4-carbonyl of the C-ring and the 5-OH on the A-ring. These results are supported by the UV-Vis interactions observed as well as previously reported crystal structures and computational studies (Bukhari et al., 2009; Pekal et al., 2011; Říha et al., 2014).

**FIGURE 5:**
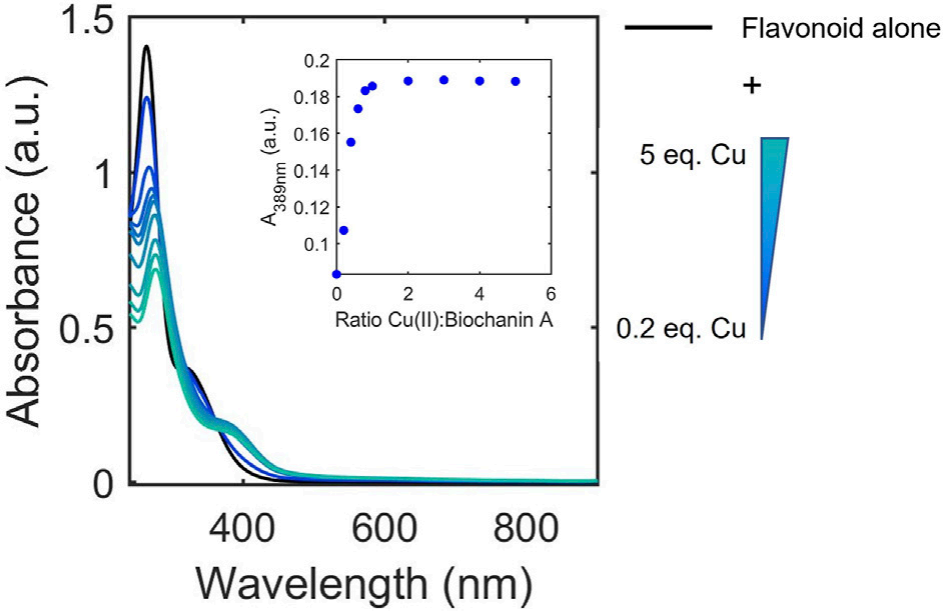
Representative electronic absorbance spectra of biochanin A upon addition of 0.2–5 equivalences of copper in 50 mM MOPS buffer (pH 7.4) for the determination of binding ratios. The inset shows absorbance at 389 nm where spectral changes occur due to Cu(II) binding to biochanin A. The changes in absorbance at 389 nm plateau at 1 equivalence of Cu(II) which indicates a 1:1 binding ratio between biochanin A and Cu(II).

The UV-Vis titrations were also examined to determine whether binding affinity classifications were associated with ligand-to-metal stoichiometries. Although the method of continuous variation (Job’s plot) has been applied to study some flavonoid/metal complexes, this method primarily works for interactions with large dissociation constants (Ulatowski et al., 2016). Thus, to allow for broader analysis of the flavonoid library, we opted to apply the mole ratio plot analysis using the characteristic absorbance of the Cu(II)-flavonoid complexes to approximate and compare binding stoichiometries of flavonoids to Cu(II) under buffered aqueous conditions at physiological pH. To achieve this, we monitored the equivalents of Cu(II) at which the intensities of the absorbance of flavonoid-Cu(II) peaks plateau, indicating stable species formation (Figure 5). Consistent with literature, the binding ratios vary between 1:2, 1:1, and 2:1 copper-to-flavonoid (Table 3). In the same way gallate modifications affects binding affinity, flavanols with gallate groups have altered binding ratios (1:1 copper-to-flavonoid) compared to their ungallated counterparts, which typically have 2:1 copper-to-flavonoid binding ratios. Additionally, as observed with the binding affinities, the O-rutinoside modification on rutin alters the binding ratio relative to its unmodified counterpart, QT, with the former having a 1:1 binding ratio in contrast to the 2:1 ratio of the latter. The 2:1 ratio of QT suggests the presence of two binding sites for Cu(II) likely occurring at the B-ring diol as well as the 4-carbonyl on the C-ring and either the 5-OH of the A-ring or the 3-OH on the C-ring. The glycone on rutin likely disrupts interactions around the 4-carbonyl allowing for binding to occur only at the B-ring. However, beyond these two observations, the binding ratios do not seem to have dependence on or correlation to structural subclass or binding affinities.

**TABLE 3:** Binding ratios of flavonoids to Cu(II) ions in solution. Ratios were determined by titrating CuSO_4_ into a 50 μM solution of flavonoid and monitoring the absorbance values of peaks that correspond to flavonoid-Cu(II) complexes.

Binding ratios (Cu:flavonoid)		
1:2	1:1	2:1
EC, Cat, hesperidin, naringin, 3-HF, luteolin, chrysin, kaempferol	EGC, ECG, EGCG, hesperetin, naringenin, rutin, cyanidin. biochanin A, genistein	QT

#### 3.1.1 Flavonoids impart antioxidant activity through multiple mechanisms

**FIGURE 6:**
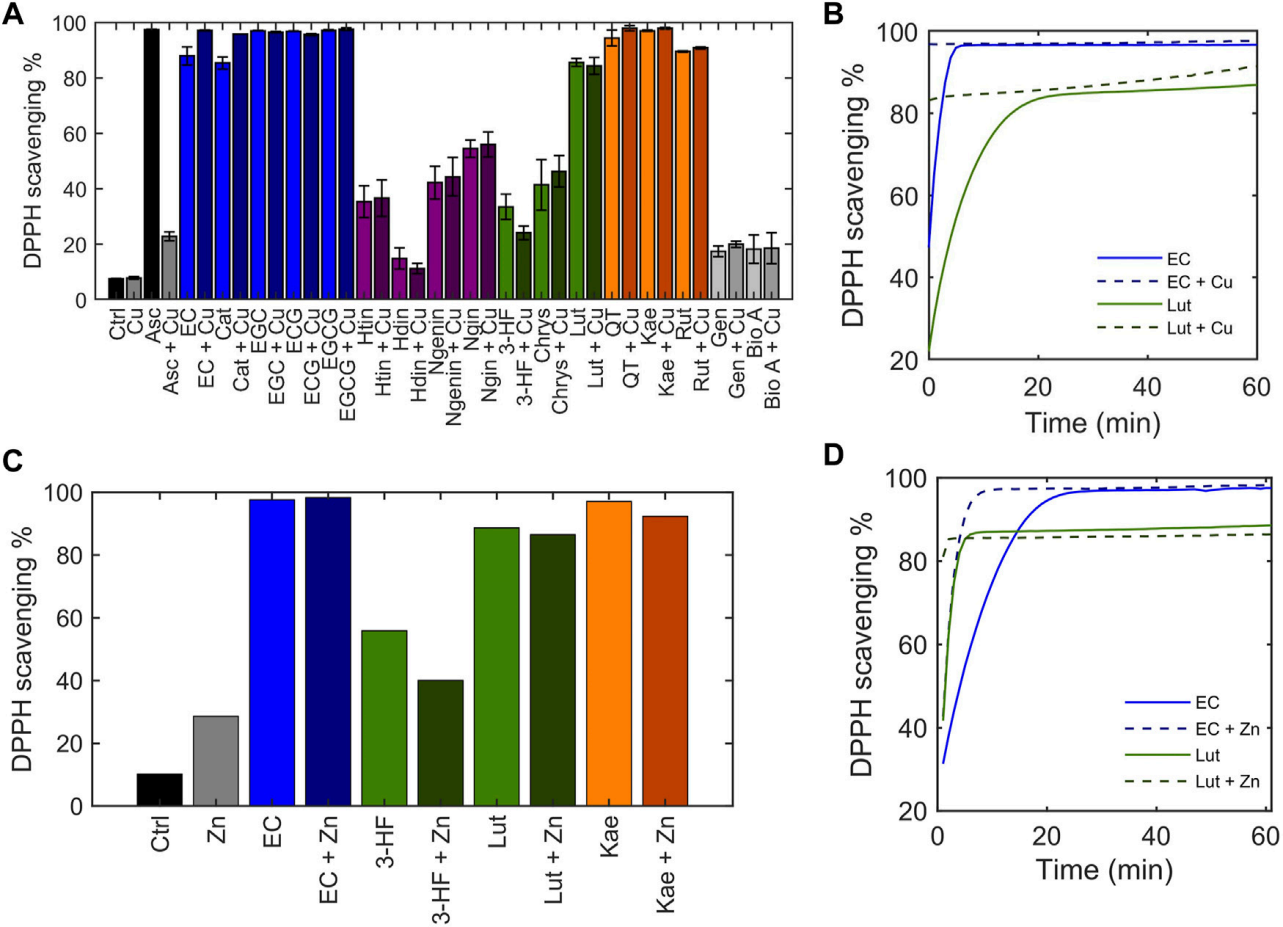
Determination of the ability of flavonoids to scavenge the DPPH radical, depicted as DPPH scavenging %. 50 μM flavonoid or ascorbic acid was incubated with 50 μM CuSO_4_ before addition of 50 μM DPPH•. **(A)** The DPPH scavenging % was determined using the 515 nm absorbance of DPPH at the end of a 1-h reaction. **(B)** Time-dependent scavenging activity of representative flavonoids EC and luteolin in the presence of 50 μM CuSO_4_ measured over the course of 1 hour. **(C)** End-point DPPH scavenging % of flavonoids in the presence of 50 μM ZnCl_2_ after a 1-h reaction. **(D)** Time-dependent DPPH scavenging activity of representative flavonoids EC and luteolin in the presence of 50 μM ZnCl_2_ measured over 1 hour.

Flavonoids are well-known to exhibit antioxidant activity, with reactive oxygen species (ROS) scavenging being one of the most common hypothesized mechanisms. The flavonoid library was classified by their antioxidant activity with the 2,2-diphenyl-1-picryl-hydrazyl-hydrate (DPPH) free radical assay (Campos et al., 2009; Xie and Schaich 2014; María Pilar de et al., 2019). The stable DPPH free radical is purple in solution, and its reduction by an antioxidation discolors the solution, allowing loss of absorbance at its λ_max_ = 515 nm to be used to assess antioxidant activity. Ascorbic acid was used as a positive control and scavenged 97% of DPPH in solution (Figure 6A). The antioxidant capacity of the flavonoids alone were tested in the absence of Cu(II) using endpoint reads of the DPPH free radical signal. The flavanols tested, all of which contain o-catechol groups in the B-ring were able to scavenge over 80% of DPPH in solution whereas the flavanones, which are similar to the flavanols but do not contain the B-ring *o-*catechol, did not exhibit DPPH radical scavenging abilities. Similarly, the flavones and isoflavones tested were unable to scavenge DPPH radical in solution except for luteolin. Of importance to note is that luteolin has an *o-*catechol group on its B-ring which the non-scavenging flavonoids lack. These results are consistent with previous studies and indicate the importance of the *o*-catechol group in antioxidant activity (Manuela Silva et al., 2002). An exception is kaempferol, which lacks the *o*-catechol group but exhibits radical scavenging abilities, suggesting that the hydroxy group on the B-ring may participate in antioxidant activity.

Little differences were observed in the endpoint reads of the DPPH assay when Cu(II) was added. While the DPPH reads at endpoint allow for general classification of the antioxidant capacity of the flavonoids, solvent effects, particularly those that may associate with the radical, have been shown to influence endpoint quantitation (Xie and Schaich 2014). A more accurate comparison on antioxidant activity can be made by monitoring scavenging kinetics. Thus, to thoroughly understand the scavenging kinetics of flavonoids in comparison to their Cu(II)-bound forms, absorbance measurements were recorded over time, beginning immediately after addition of DPPH to solution. For all flavonoids that exhibit DPPH radical scavenging properties based on endpoint reads, the presence of Cu(II) increased the kinetics of the scavenging reaction (representative plots of EC and luteolin shown in Figure 6B). It has been suggested that rather than the antioxidant activity occurring from redox reactions with the Cu(II) center, coordination of Cu(II) at the hydroxy groups on the B-ring stabilizes a semiquinone radical intermediate facilitating the DPPH radical scavenging mechanism (Bukhari et al., 2009). To test this hypothesis, Zn(II) was substituted for Cu(II). Zn(II), like Cu(II), behaves as a Lewis acid, but it is redox-inactive. The scavenging activities of flavonoids in the presence of Zn(II) show similar trends as in the presence of Cu(II) (Figures 6C,D). The rate of scavenging was increased in the presence of Zn(II) which supports the hypothesis that the increased antioxidant activity with Cu(II) addition is due to its interaction with the B-ring hydroxy groups as a Lewis acid rather than metal-centered redox activity. The DPPH assay demonstrates that flavonoids’ radical scavenging abilities are affected by Cu(II) but are not correlated with the strength of copper interaction.

**FIGURE 7:**
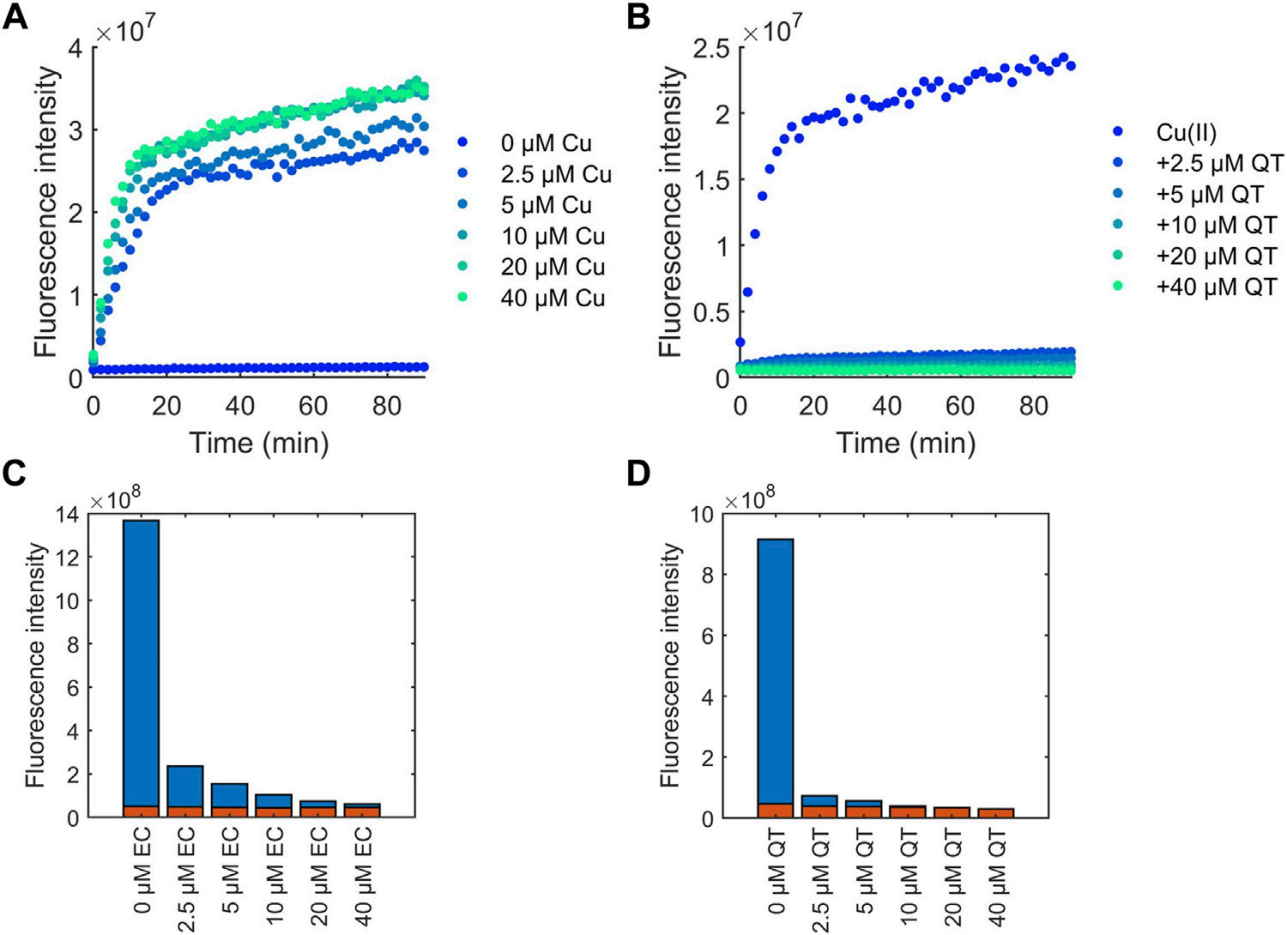
CCA fluorescence intensity measurements for determining the effects of flavonoids on the time-dependent generation of ^•^OH. Flavonoid effects were compared to **(A)** Cu(II) addition alone, with **(B)** QT shown as an example. 2.5 mM CCA and 50 μM ascorbic acid were used for all experiments. The calculated area under the curve over the course of 90 min was measured for flavonoid solutions. **(C)** EC and **(D)** QT effects are shown as representative plots in the presence (blue) and absence (red) of Cu(II).

Another mechanism by which flavonoids potentially possess antioxidant activity is by chelating redox-active Cu(II) ions thereby preventing production of ROS produced via Fenton-like chemistry (Hu et al., 2016). To assess this, coumarin-3-carboxylic (3-CCA) was used as a probe to examine the protection of flavonoids against Cu(II)-induced production of ^•^OH (Hu et al., 2016). Ascorbic acid and copper undergo a redox cycling reaction which produces ^•^OH. 3-CCA reacts with ^•^OH to form 7-hydroxy-3-carboxycoumarinic acid which fluoresces with excitation at 388 nm and emission at 450 nm. The fluorescence intensity can be monitored to observe production of ^•^OH in solution. As expected, the concentration of Cu(II) ions in solution was positively correlated to the amount of ^•^OH detected (Figure 7A). Introduction of all flavonoids to solution decreased detected ^•^OH, with increasing amounts of flavonoids decreasing ^•^OH production (Figures 7B; Supplementary Figure S2). In contrast to the flavonoid effects on DPPH scavenging, the protective effects of the flavonoids against Cu(II)-induced production of ROS correlates to their binding affinities. For example, at 4-fold excess, EC is unable to reduce production of ^•^OH to baseline levels (Figure 7C), whereas QT was able to stop ^•^OH generation at 1 equivalent (Figure 7D).

Flavonoids are often touted and have been extensively studied for their antioxidant effects. Due to their known interactions with copper ions, it has been hypothesized that some of their antioxidant activity is a result of those interactions (Fernandez et al., 2002; Ren et al., 2008; Pekal et al., 2011; Cherrak et al., 2016). The discrepancies between the CCA and DPPH assays indicate that while copper interactions do have an effect on the antioxidant activity of flavonoids, the mechanism by which copper has its influence varies based on the flavonoid. While copper binding affinity generally correlates with ^•^OH levels from Cu(II)-induced Fenton-like chemistry as measured with the CCA assay, these trends do not apply to general free radical scavenging as measured with the DPPH assay. It is important to note that the ability to protect against ^•^OH generation is not directly correlated to structure of the flavonoids, but rather to the binding affinities of the flavonoids to Cu(II). Stronger binders, like QT and kaempferol, can prevent ^•^OH production, whereas weaker binders, like EC, cannot eliminate ^•^OH generation. Both the CCA and DPPH assays demonstrate that antioxidant activity of flavonoids is not delineated by subclass but rather functional group location and identity.

### 3.2 Understanding the bioactivity of flavonoids

#### 3.2.1 Effects of flavonoids on copper trafficking in yeast

**FIGURE 8:**
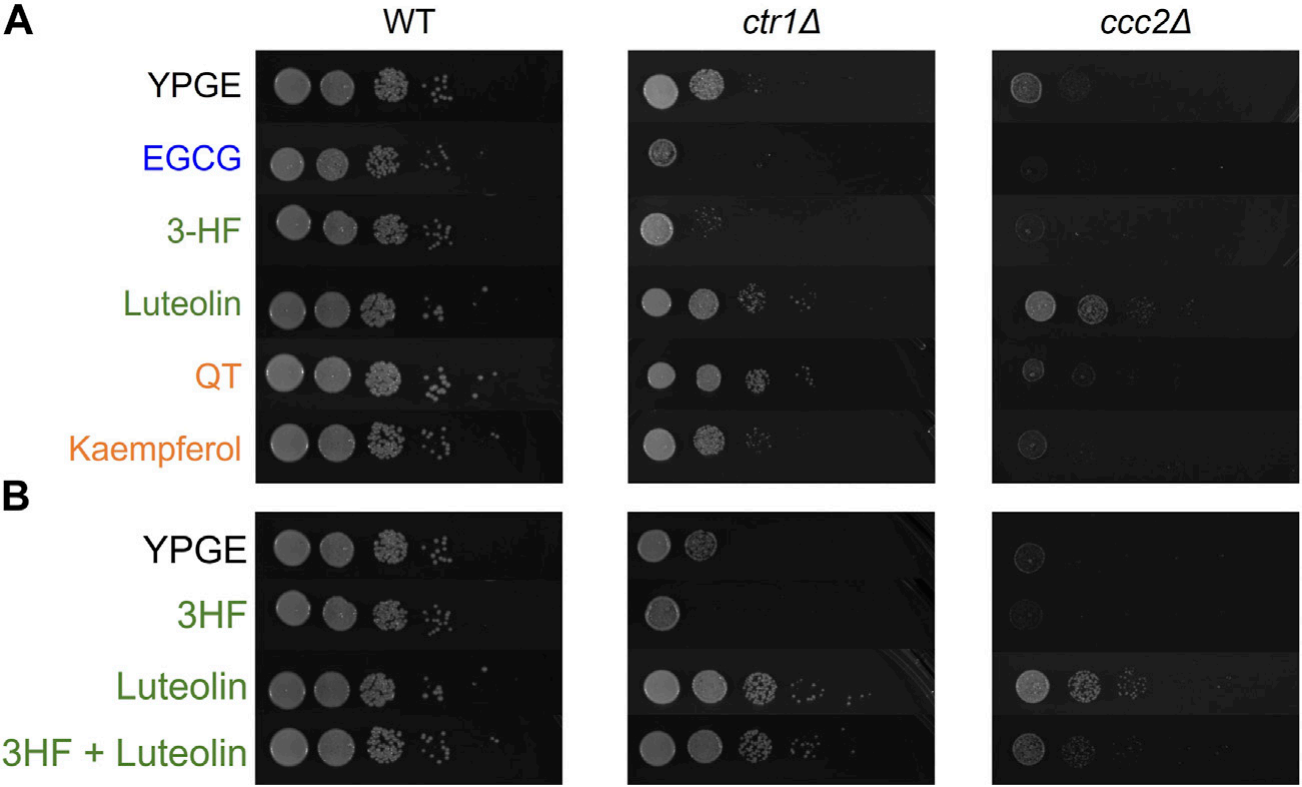
Serially diluted *S. cerevisiae* strains were spotted on the noted YPGE plates and incubated at 37°C for 4 days before imaging. **(A)** YPGE agar plates were prepared with 25 μM of the noted flavonoid. **(B)** Mixed ligand plates were prepared with 25 μM of each noted flavonoid. Treatment with mixed ligands exhibit changes suggestive of competition between ligands.

Having gained an understanding of the structural features important to flavonoid-copper interactions in solution, we extended investigations to probe how the flavonoids’ appreciable interactions with Cu(II) in buffered solutions influence copper trafficking in cell-based models. The ability of these flavonoids to transport copper was probed in *Saccharomyces cerevisiae* (*S. cerevisiae*) as a model eukaryotic organism (Soma et al., 2018). Two knockout strains of *S. Cerevisiae*, *ctr1Δ* and *ccc2Δ*, were treated with various flavonoids to observe differences in growth. The *ctr1* gene encodes a high-affinity copper transporter localized at the plasma membrane, and its knockdown restricts copper import from the extracellular space into the cytosol (Wu et al., 2009). The *ccc2* gene encodes a copper-transporting ATPase hypothesized to translocate copper from the cytosol to extracytosolic compartments, and its knockdown has been shown to induce a functional copper deficiency (Yuan et al., 1995). Under aerobic growth conditions (e.g. YPGE media), compared to its wild-type (WT) counterpart, the knockout strains show impaired growth that is rescued with copper supplementation (Supplementary Figure S3). We posited that copper-binding flavonoids could affect copper bioavailability to the knockdown strains, which could be monitored by differences in yeast growth. Specifically, we investigated the activity of 3-HF, luteolin, QT, and kaempferol, as these represent strong binders with a common ring conjugation structure. Growth rescue of *ctr1Δ* cells would suggest that copper is being imported by the flavonoid while rescue of *ccc2Δ* cells would indicate that the flavonoid can improve intracellular copper availability to extracytosolic compartments. To assess growth with a large dynamic range of detection, a spot assay was performed, where yeast were plated on agar in a 1:10 dilution series of four dilutions. Interestingly, despite the similarities of the flavonoids with respect to solution-based binding affinities and ring structures, they showed notable differences in effects on growth rescue of the knockouts (Figure 8).

In both knockout strains, 3-HF further restricts growth*,* which may indicate chelation of extracellular copper without transport into the cell. Conversely, luteolin treatment exhibits growth rescue effects on both *ctr1Δ* and *ccc2Δ* suggesting that it can transport copper into the cell and affect its intracellular availability. The differences in the behavior of these two may be attributed in their relative hydrophobicities. *S. cerevisiae* have cell walls that are hydrophilic (Klis et al., 2002). The relatively higher hydrophobicity of 3-HF (reported log *p* = 4.17 (Pogodaeva et al., 2012)) may explain its inability to transport copper into yeast cells in contrast to the more hydrophilic luteolin [reported log *p* = 0.7 (Quintieri et al., 2008)] (Supplementary Table S1). The simultaneous treatment with both 3-HF and luteolin result in a combined effect, with growth rescue that is less than luteolin but similar to copper supplementation alone suggesting that the two flavonoids may compete in their effects (Figure 8B). This may point to the possibility of modulation of copper ionophoric effects based on flavonoid content. Kaempferol and QT have reported log *p* values that are similar to one another but are in between those of luteolin and 3-HF (log *p* = 1.87 (Sreelakshmi et al., 2017) and 1.82 (Rothwell et al., 2005) respectively). Interestingly, while kaempferol shows only modest to no growth rescue of the *ctr1Δ* and *ccc2Δ,* QT shows a strong growth rescue of the *ctr1Δ* strain comparable to that of luteolin. This may be in part due to the ability of QT to complex Cu(II) at a 2:1 ratio of copper-to-flavonoid, allowing for translocation of higher concentrations of copper into the cell. The growth rescue by both luteolin and QT of the *ctr1Δ* strain may also point to the importance of the catechol in the B-ring in facilitating copper import. However, in the *ccc2Δ* strain, no growth rescue is observed by QT, suggesting that while QT may facilitate copper import, it does not have a beneficial impact on extracytosolic copper availability.

Additionally, we tested the effects of EGCG on the growth of the knockout strains, as EGCG is a well-reported copper modulator in cell-based assays. In both strains, EGCG exhibited potent inhibition on cell growth, suggesting that it may serve as an extracellular Cu(II) chelator to withhold the metal ion from the organism.

#### 3.2.2 Effects of flavonoids on copper trafficking in human hepatocytes

**FIGURE 9:**
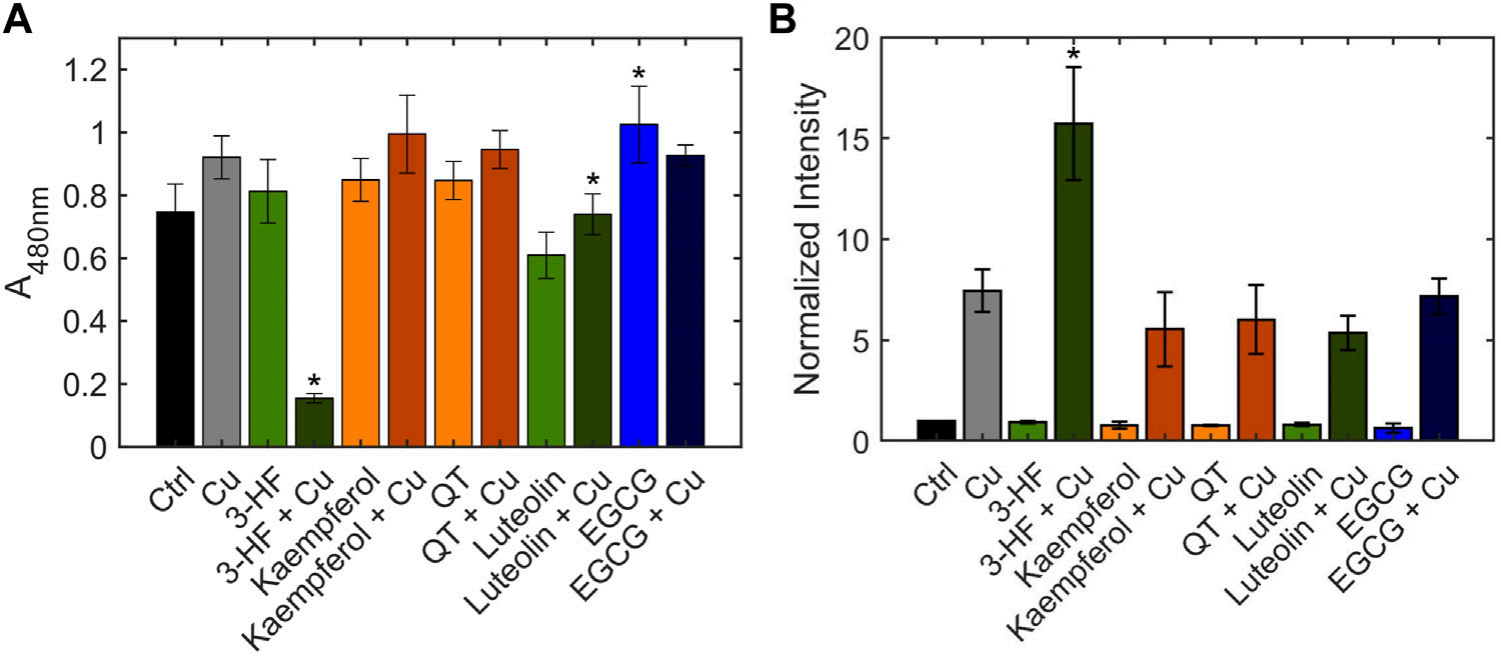
**(A)** Cell viability of HepG2 cells in the presence and absence of flavonoids and Cu(II), measured using the MTS assay. HepG2 cells were stimulated with 20 μM flavonoid with or without 50 μM CuSO_4_. The MTS reagent was added after 24 h. After 1 h incubation with the reagent, absorbance was measured at 490 nm. **(B)** Quantification of total cellular copper of HepG2 cells in the presence and absence of flavonoids and Cu(II), measured by ICP-OES. HepG2 cells were stimulated with 20 μM flavonoid with or without 50 μM CuSO_4_ for 24 h. Error bars represent SD, *n* = 3. *p < 0.05 versus the vehicle control.

**FIGURE 10:**
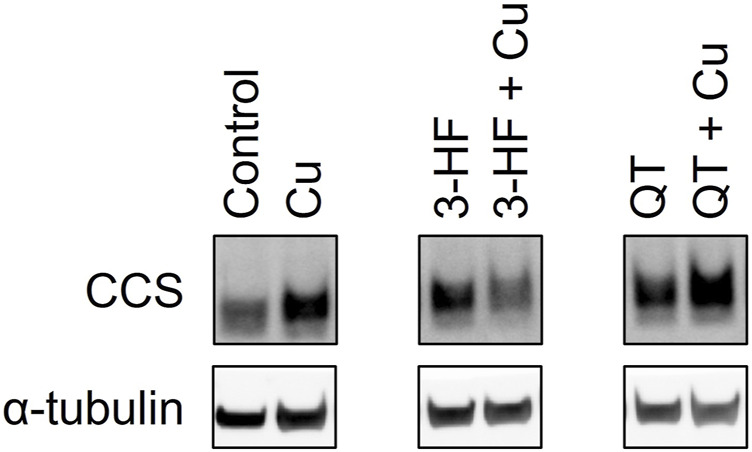
Western blot analysis of the CCS protein in lysates of HepG2 cells in the presence and absence of the flavonoids 3-HF or QT and Cu(II). HepG2 cells were stimulated with 20 μM flavonoid with and without 50 μM CuSO_4_ and incubated for 24 h. Cell lysates were collected, and Western blot analysis was performed using antibodies specific for CCS with α-tubulin as a control. Western blot images for the remaining assessed flavonoids are shown in Supplementary Figure S5.

The inability of 3-HF to improve copper availability to the yeast knockout strains was a surprising observation, given that recent work reported that this particular flavonoid acts as a potent copper ionophore in mammalian cancer cell lines. In this context, Dai et al. posited that the delivery of copper by 3-HF could cause a redox imbalance in the cell, leading to copper-induced cell death (Dai et al., 2017), or “cuproptosis” (Oliveri 2022). We thus assessed the same flavonoids in HepG2 cells, a human hepatocarcinoma cell line sensitive to cuproptosis. Cell viability was measured using the MTS assay in which an MTS tetrazolium compound is reduced by the mitochondria of viable cells to produce a colored formazan complex whose absorbance is monitored. Consistent with the previous report, our results show that in the presence of supplemented copper, 3-HF significantly reduces cell viability (Figure 9A), and this effect correlates with increased intracellular copper levels, as measured by ICP-OES (Figure 9B). Conversely to yeast cells, HepG2 cells have a hydrophobic cell membrane, which may explain the differential effects in copper import between the two systems. The effect of 3-HF on copper metabolism is further validated by changes to the expression of the copper chaperone for superoxide dismutase (CCS), a reported marker for cytosolic copper deficiency (Bertinato and L’Abbé 2003). Interestingly, supplemental copper increases CCS expression, while the addition of 3-HF with supplemental copper decreases expression (Figure 10). Although CCS expression has been shown to increase in the presence of copper chelators and copper-deficient conditions, its response to acute treatment of supplemental copper remains unclear. While this complicates the interpretation of the CCS expression data, the difference between supplemented copper with and without 3-HF remain apparent, suggesting that the flavonoid modulates copper by different mechanisms than treatment with Cu(II) salt alone.

While none of the other flavonoids tested significantly altered intracellular copper levels relative to the no-flavonoid controls, luteolin and EGCG treatments signficantly affected cell viability. Previous work has shown that while EGCG shows antioxidant properties, its copper complex conversely functions as a pro-oxidant (Azam et al., 2004). These contrasting effects may be reflected in the cell viability data where EGCG improves cell viability over the control whereas the presence of Cu(II) reduces this effect. This may suggest the differences in cell viability with EGCG may be due to affecting the reactive oxygen species balance in the cell rather than by copper-modulating mechanisms. Luteolin, on the other hand, reduces cell viability, and this effect is attenuated by Cu(II) addition. Given the relative hydrophilicity of luteolin, it is possible that the flavonoid alters reactive oxygen species balance in the extracellular environment, but further studies are required to assess this effect. However, as both EGCG and luteolin treatments show no significant changes in either intracellular copper levels or CCS levels, their impact on cell viability may be independent of copper modulation. In contrast, while QT shows no changes in intracellular copper levels nor cell viability, the flavonoid induces a notable increase in expression in CCS in the presence of Cu(II). This may suggest that while QT does not affect copper import, it may modulate intracellular copper distribution and availability.

The cell-based assays demonstrate that despite similar behaviors of the flavonoids in solution, these are not necessarily predictive of their *biological* behavior. Moreover, even among eukaryotic systems, the structural features of the flavonoids may lend to differences in interactions in components such as membrane environments.

## 4 Conclusion

In this work, we focused on characterizing and assessing the ability of flavonoids to serve as modulators of copper trafficking. Structure-based characterization of flavonoid-Cu(II) interactions in buffered aqueous solutions showed wide diversity in the binding affinities and ratios that correspond more closely to specific functional group modifications and locations rather than the traditional subclassification of flavonoids by ring structures. Binding affinities correlated to protective effects against ^•^OH generation but not necessarily general scavenging activity. We further assessed how structural factors may affect the ability of flavonoids with appreciable Cu(II) affinity to traffic the metal in biological contexts. In yeast models, we found that hydrophobicities trend with the transport of copper into the cells. The more hydrophilic flavonoids, luteolin and QT, can cross the yeast cell wall and rescue growth of strains with copper trafficking proteins knocked out. However, a different trend was observed in mammalian HepG2 cells, which have relatively hydrophobic cell membranes, with the most pronounced copper transport effects exhibited by 3-HF. 3-HF increased intracellular copper levels and subsequently induced cuproptosis. While the exact mechanisms of 3-HF transport require further elucidation, decreased CCS protein expression suggests that 3-HF affects cellular copper trafficking mechanisms. Though 3-HF had the most marked effect on copper trafficking markers, treatment with QT also demonstrated effects on CCS expression suggesting potential modulation of intracellular localization. Taken together, our studies demonstrate an approach for linking the structure of natural products to their potential functions as copper modulators. Future work should take into account organism-specific copper modulation as well as the heightened importance of functional group substitution and hydrophobicities rather than traditional subclassifications. The data offers insight to guiding principles for identifying such agents within the flavonoid structural family, which can both inform nutritional recommendations as well as therapeutic agent design for impacting copper-associated disorders.

## Data Availability

The raw data supporting the conclusions of this article will be made available by the authors, without undue reservation.

## Abbreviations

3-HF, 3-hydroxyflavone 3-hydroxy-2-phenylchromen-4-one; Bio A, Biochanin A 5,7-dihydroxy-3-(4-methoxyphenyl)chromen-4-one; Cat, (±)-catechin (2S,3R)-2-(3,4-dihydroxyphenyl)-3,4-dihydro-2H-chromene-3,5,7-triol; Chrys, Chrysin 5,7-dihydroxy-2-phenylchromen-4-one; Cyan, Cyanidin 2-(3,4-dihydroxyphenyl)chromenylium-3,5,7-triol; EC, (-)-epicatechin (2*R*, 3*R*)-2-(3,4-dihydroxyphenyl)-3,4-dihydro-2*H*-chromene-3,5,7-triol; ECG, (-)-epicatechin gallate [(2R,3R)-2-(3,4-dihydroxyphenyl)-5,7-dihydroxy-3,4-dihydro-2H-chromen-3-yl] 3,4,5-trihydroxybenzoate; EGC, (-)-epigallocatechin (2R,3R)-2-(3,4,5-trihydroxyphenyl)-3,4-dihydro-2H-chromene-3,5,7-triol; EGCG, (-)-epigallocatechin gallate [(2R,3R)-5,7-dihydroxy-2-(3,4,5-trihydroxyphenyl)-3,4-dihydro-2H-chromen-3-yl] 3,4,5-trihydroxybenzoate; Gen, Genistein 5,7-dihydroxy-3-(4-hydroxyphenyl)chromen-4-one; Hdin, Hesperidin (2S)-5-hydroxy-2-(3-hydroxy-4-methoxyphenyl)-7-[(2S,3R,4S,5S,6R)-3,4,5-trihydroxy-6-[[(2R,3R,4R,5R,6S)-3,4,5-trihydroxy-6-methyloxan-2-yl]oxymethyl]oxan-2-yl]oxy-2,3-dihydrochromen-4-one; Htin, Hesperetin (2S)-5,7-dihydroxy-2-(3-hydroxy-4-methoxyphenyl)-2,3-dihydrochromen-4-one; Kae, Kaempferol 3,5,7-trihydroxy-2-(4-hydroxyphenyl)chromen-4-one; Lut, Luteolin 2-(3,4-dihydroxyphenyl)-5,7-dihydroxychromen-4-one; Ngenin, Naringenin (2S)-5,7-dihydroxy-2-(4-hydroxyphenyl)-2,3-dihydrochromen-4-one; Ngin, Naringin (2S)-7-[(2S,3R,4S,5S,6R)-4,5-dihydroxy-6-(hydroxymethyl)-3-[(2S,3R,4R,5R,6S)-3,4,5-trihydroxy-6-methyloxan-2-yl]oxyoxan-2-yl]oxy-5-hydroxy-2-(4-hydroxyphenyl)-2,3-dihydrochromen-4-one; QT, Quercetin 2-(3,4-dihydroxyphenyl)-3,5,7-trihydroxychromen-4-one; Rut, Rutin 2-(3,4-dihydroxyphenyl)-5,7-dihydroxy-3-[(2S,3R,4S,5S,6R)-3,4,5-trihydroxy-6-[[(2R,3R,4R,5R,6S)-3,4,5-trihydroxy-6-methyloxan-2-yl]oxymethyl]oxan-2-yl]oxychromen-4-one.

## References

[B1] Anderson S. N. Larson M. T. Berreau L. M. (2016). Solution or solid-it doesn’t matter: Visible light-induced CO release reactivity of zinc flavonolato complexes. Dalton Trans. 45 (37), 14570–14580. 10.1039/c6dt01709f 27711794

[B2] Ansari A. A. (2008). DFT and 1H NMR molecular spectroscopic studies on biologically anti-oxidant active paramagnetic lanthanide(III)-Chrysin complexes. Main. Group Chem. 7 (1), 43–56. 10.1080/10241220801912637

[B3] Atp A. (1979). Metal ion/buffer interactions. Eur. J. Biochem. 530, 523–530.

[B4] Azab H. A. El-Nady A. M. (1994). Ternary complexes in solution. Comparison of the coordination tendency of some biologically important zwitterionic buffers toward the binary complexes of Cu(II) and adenosine 5′-Mono-5′-Di-and 5′-triphosphate. Monatsh. Chem. 125 (8–9), 849–858. 10.1007/BF00812698

[B5] Azab H. A. Orabi A. S. Enas T. (2001). Role of biologically important zwitterionic buffer secondary ligands on the stability of the mixed-ligand complexes of divalent metal ions and adenosine 5′-Mono-5′-Di-and 5′-triphosphate. J. Chem. Eng. Data 46 (2), 346–354. 10.1021/je0001779

[B6] Azam S. Hadi N. Khan N. U. Hadi S. M. (2004). Prooxidant property of green tea polyphenols epicatechin and epigallocatechin-3-gallate: Implications for anticancer properties. Toxicol. Vitro 18 (5), 555–561. 10.1016/j.tiv.2003.12.012 15251172

[B7] Baldari Silvia Di Rocco G. Toietta G. (2020). Current biomedical use of copper chelation therapy. Int. J. Mol. Sci. 21 (3), 1069. 10.3390/ijms21031069 PMC7037088 32041110

[B8] Bertinato J. L’Abbé M. R. (2003). Copper modulates the degradation of copper chaperone for Cu, Zn superoxide dismutase by the 26 S proteosome. J. Biol. Chem. 278 (37), 35071–35078. 10.1074/jbc.M302242200 12832419

[B9] Bukhari S. B. Memon S. Mahroof-Tahir M. Bhanger M. I. (2009). Synthesis, characterization and antioxidant activity copper-quercetin complex. Spectrochimica Acta Part A Mol. Biomol. Spectrosc. 71 (5), 1901–1906. 10.1016/j.saa.2008.07.030 18783981

[B10] Campos C. Guzmán R. López-Fernández E. Casado Á. (2009). Evaluation of the copper(II) reduction assay using bathocuproinedisulfonic acid disodium salt for the total antioxidant capacity assessment: The CUPRAC-BCS assay. Anal. Biochem. 392 (1), 37–44. 10.1016/j.ab.2009.05.024 19464250

[B11] Cherrak S. A. Mokhtari-Soulimane N. Berroukeche F. Bensenane B. Cherbonnel A. Merzouk H. (2016). *In vitro* antioxidant versus metal ion chelating properties of flavonoids: A structure-activity investigation. PLoS ONE 11 (10), e0165575. 10.1371/journal.pone.0165575 27788249 PMC5082868

[B12] Cremonini E. Wang Z. Ahmed B. Adamo A. M. Daveri E. Mills D. A. (2018). (-)-Epicatechin protects the intestinal barrier from high fat diet-induced permeabilization: Implications for steatosis and insulin resistance. Redox Biol. 14 (2017), 588–599. 10.1016/j.redox.2017.11.002 29154190 PMC5691220

[B13] Dai F. Wen J. Y. Yu T. D. Zhen Bao X. Zhuang Li X. Zhou B. (2017). Structural basis, chemical driving forces and biological implications of flavones as Cu(II) ionophores. Free Radic. Biol. Med. 108, 554–563. 10.1016/j.freeradbiomed.2017.04.023 28431962

[B14] Datta N. Singanusong R. Chen S. S. Yao L. H. Jiang Y. M. Shi J. (2004). Flavonoids in food and their health benefits. Plant Foods Hum. Nutr. 59, 113–122. 10.1007/s11130-004-0049-7 15678717

[B15] Davis A. L. Ya C. Davies A. P. Lewis J. R. (1996). 1H and 13C NMR assignments of some green tea polyphenols. Magn. Reson. Chem. 34 (11), 887–890. 10.1002/(SICI)1097-458X(199611)34:11<887::AID-OMR995>3.0.CO;2-U

[B16] Delius J. Frank O. Hofmann T. (2017). Label-free quantitative 1H NMR spectroscopy to study low-affinity ligand–protein interactions in solution: A contribution to the mechanism of polyphenol-mediated astringency. PLoS ONE 12 (9), e0184487. 10.1371/journal.pone.0184487 28886151 PMC5590944

[B17] Escandar G. M. Sala L. F. (1991). Complexing behavior of rutin and quercetin. Can. J. Chem. 69 (12), 1994–2001. 10.1139/v91-288

[B18] Fang X. Gao W. Yang Z. Gao Z. Li H. (2020). Dual anti-/prooxidant behaviors of flavonoids pertaining to Cu(II)-Catalyzed tyrosine nitration of the insulin receptor Kinase domain in an antidiabetic study. J. Agric. Food Chem. 68 (22), 6202–6211. 10.1021/acs.jafc.0c01676 32395994

[B19] Fernandez M. T. Mira M. L. Helena Florêncio M. Jennings K. R. (2002). Iron and copper chelation by flavonoids: An electrospray mass spectrometry study. J. Inorg. Biochem. 92 (2), 105–111. 10.1016/S0162-0134(02)00511-1 12459155

[B20] Hu X. Zhang Q. Wang W. Yuan Z. Zhu X. Chen B. (2016). Tripeptide GGH as the inhibitor of copper-amyloid-β-mediated redox reaction and toxicity. ACS Chem. Neurosci. 7 (9), 1255–1263. 10.1021/acschemneuro.6b00145 27433833

[B21] Hunsaker E. W. Franz K. J. (2019). Emerging opportunities to manipulate metal trafficking for therapeutic benefit. Inorg. Chem. 1, 13528–13545. 10.1021/acs.inorgchem.9b01029 PMC7272113 31247859

[B22] Kandegedara A. Rorabacher D. B. (1999). Noncomplexing tertiary amines as ‘better’ buffers covering the range of PH 3-11. Temperature dependence of their acid dissociation constants. Anal. Chem. 71 (15), 3140–3144. 10.1021/ac9902594 21662904

[B23] Klis Frans M. Mol P. Hellingwerf K. Brul S. (2002). Pieternella mol, klaas hellingwerf, and stanley BrulDynamics of cell wall structure in Saccharomyces cerevisiae. FEMS Microbiol. Rev. 26 (3), 239–256. 10.1111/j.1574-6976.2002.tb00613.x 12165426

[B24] Korotkova E. I. Voronova O. A. Dorozhko E. V. (2012). Study of antioxidant properties of flavonoids by voltammetry. J. Solid State Electrochem. 16 (7), 2435–2440. 10.1007/s10008-012-1707-6

[B25] Kumar S. Pandey A. K. (2013). Chemistry and biological activities of flavonoids: An overview. Sci. World J. 2013, 162750. Availble at: http://www.pubmedcentral.nih.gov/articlerender.fcgi?artid=3891543&tool=pmcentrez&rendertype=abstract . 10.1155/2013/162750 PMC3891543 24470791

[B26] Manuela Silva M. Santos M. R. Caroco G. Rocha R. Goncalo J. Mira L. (2002). Structure-antioxidant activity relationships of flavonoids: A Re-examination. Free Radic. Res. 36 (11), 1219–1227. 10.1080/198-1071576021000016472 12592674

[B27] María Pilar de T. Yolanda Cavero R. Isabel Calvo M. Vizmanos J. L. W. (2019). A simple and a reliable method to quantify antioxidant activity *in vivo* . Antioxidants 8 (5), 142. 10.3390/antiox8050142 PMC6562907 31121854

[B28] Oliveri V. (2022). Selective targeting of cancer cells by copper ionophores: An overview. Front. Mol. Biosci. 9, 841814. 10.3389/fmolb.2022.841814 35309510 PMC8931543

[B29] Pȩkal A. Biesaga M. Pyrzynska K. (2011). Interaction of quercetin with copper ions: Complexation, oxidation and reactivity towards radicals. BioMetals 24 (1), 41–49. 10.1007/s10534-010-9372-7 20835752

[B30] Perron N. R. García C. R. Pinzón J. R. Chaur M. N. Brumaghim J. L. Brumaghim J. L. (2011). Antioxidant and prooxidant effects of polyphenol compounds on copper-mediated DNA damage. J. Inorg. Biochem. 105 (5), 745–753. 10.1016/j.jinorgbio.2011.02.009 21481816

[B31] Pogodaeva N. N. Medvedeva S. A. Sukhov B. G. Larina L. I. (2012). Spectroscopic study of the reaction of a natural arabinogalactan Polysaccharide with 3-hydroxyflavones in aqueous solutions. Chem. Nat. Compd. 48 (5), 723–727. 10.1007/s10600-012-0368-0

[B32] Quintieri L. Palatini P. Nassi A. Ruzza P. Floreani M. (2008). Flavonoids Diosmetin and luteolin Inhibit Midazolam metabolism by human liver Microsomes and recombinant CYP 3A4 and CYP3A5 Enzymes. Biochem. Pharmacol. 75 (6), 1426–1437. 10.1016/j.bcp.2007.11.012 18191104

[B33] Ren J. Meng S. Lekka C. E. Kaxiras E. (2008). Complexation of flavonoids with iron: Structure and optical signatures. J. Phys. Chem. B 112 (6), 1845–1850. 10.1021/jp076881e 18211058

[B34] Rice-Evans C. A. Miller N. J. George P. (1996). Structure-antioxidant activity relationships of flavonoids and phenolic acids. Free Radic. Biol. Med. 20 (7), 933–956. 10.1016/0891-5849(95)02227-9 8743980

[B35] Říha M. Jana K. Filipský T. Macáková K. Rocha L. Bovicelli P. (2014). *In vitro* evaluation of copper-chelating properties of flavonoids. RSC Adv. 4 (62), 32628–32638. 10.1039/c4ra04575k

[B36] Rothwell J. A. Day A. J. Morgan M. R. A. (2005). Experimental determination of octanol-water partition Coefficients of quercetin and related flavonoids. J. Agric. Food Chem. 53 (11), 4355–4360. 10.1021/jf0483669 15913295

[B37] Samsonowicz M. Regulska E. (2017). Spectroscopic study of molecular structure, antioxidant activity and biological effects of metal hydroxyflavonol complexes. Spectrochimica Acta Part A Mol. Biomol. Spectrosc. 173, 757–771. 10.1016/j.saa.2016.10.031 27792987

[B38] Soma S. Latimer A. J. Chun H. Vicary A. C. Timbalia S. A. Boulet A. (2018). Elesclomol restores mitochondrial function in genetic models of copper deficiency. Proc. Natl. Acad. Sci. U. S. A. 115 (32), 8161–8166. 10.1073/pnas.1806296115 30038027 PMC6094114

[B39] Sreelakshmi V. Raj N. Abraham A. (2017). Evaluation of the drug-like properties of kaempferol, Chrysophanol and Emodin and their interactions with EGFR tyrosine Kinase - an *in silico* approach. Nat. Product. Commun. 12 (6), 915–920. 10.1177/1934578x1701200621

[B40] Ulatowski F. Dabrowa K. Bałakier T. Jurczak J. (2016). Recognizing the limited applicability of job plots in studying host-guest interactions in supramolecular chemistry. J. Org. Chem. 81 (5), 1746–1756. 10.1021/acs.joc.5b02909 26866984

[B41] Weber G. (1988). HPLC with electrochemical detection of metal-flavonoid-complexes isolated from food. Chromatographia 26 (1), 133–138. 10.1007/BF02268137

[B42] Wu X. Sinani D. Kim H. Lee J. (2009). Copper transport activity of yeast Ctr1 is down-regulated via its C terminus in response to excess copper. J. Biol. Chem. 284 (7), 4112–4122. 10.1074/jbc.M807909200 19088072 PMC2640970

[B43] Xie J. Schaich K. M. (2014). Re-evaluation of the 2, 2-diphenyl-1-picrylhydrazyl free radical (DPPH) assay for antioxidant activity. J. Agric. Food Chem. 62 (19), 4251–4260. 10.1021/jf500180u 24738928

[B44] Xu Y. Yang J. Lu Y. Qian L.-L. Yin Yang Z. Min Han R. (2020). Copper(II) coordination and translocation in luteolin and effect on radical scavenging. J. Phys. Chem. B 124 (2), 380–388. 10.1021/acs.jpcb.9b10531 31845805

[B45] Yuan D. S. Stearman R. Dancis A. Dunn T. Beeler T. Klausner R. D. (1995). The menkes/wilson disease gene homologue in yeast provides copper to a ceruloplasmin-like oxidase required for iron uptake. Proc. Natl. Acad. Sci. U. S. A. 92 (7), 2632–2636. 10.1073/pnas.92.7.2632 7708696 PMC42272

[B46] Zhang L. Liu Y. Wang Y. Xu M. Hu X. (2018). UV–Vis spectroscopy combined with chemometric study on the interactions of three dietary flavonoids with copper ions. Food Chem. 263, 208–215. 10.1016/j.foodchem.2018.05.009 29784309

[B47] Zhao B. Wang X. Liu H. Lv C. Lu J. (2020). Structural characterization and antioxidant activity of oligosaccharides from panax ginseng C. A. Meyer. Int. J. Biol. Macromol. 150, 737–745. 10.1016/j.ijbiomac.2020.02.016 32027898

