# Supplementary material for: Structure-activity assessment of flavonoids as modulators of copper transport
Source: Front Chem. 2022 Aug 23;10:972198. doi: 10.3389/fchem.2022.972198 (PMC9445161; doi:10.3389/fchem.2022.972198)
Supplement: Supplementary file 1 [file DataSheet1.pdf]

Supplementary Information

SI Figure 1.

Flavonoid	MOPS (-log K = 4) K_d = 100 µM	ACES (-log K = 4.76) K_d = 17.4 µM	BisTris (-log K = 5.27) K_d = 5.3 µM	Tricine (-log K = 7.3) K_d = 50 nM
EC				
Cat				
EGC				
ECG				
ECGC				
QT				
Hesperetin				
3-HF				

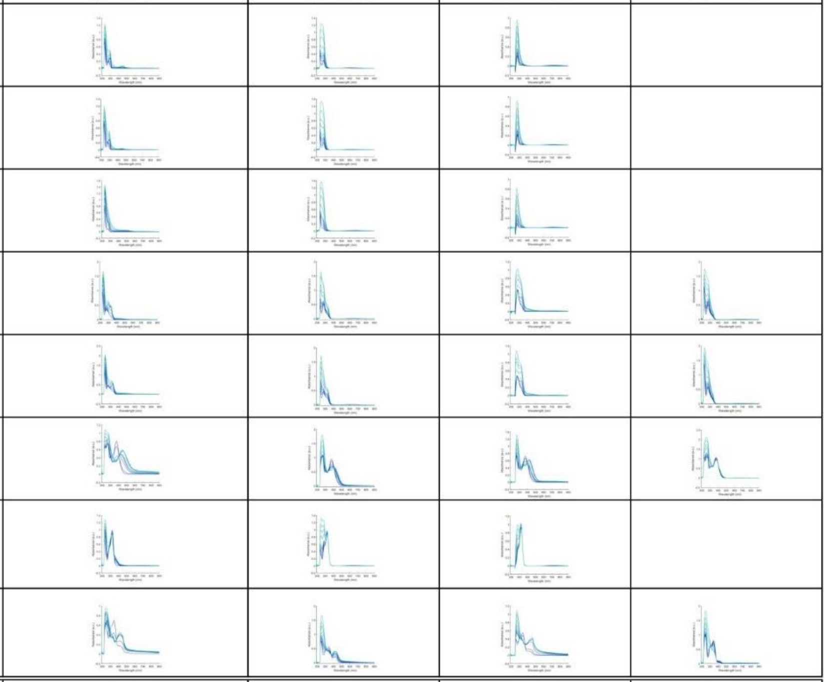

Flavonoid	MOPS (log K = 4)	ACES (log K = 4.76)	BisTris (log K = 5.27)	Tricine (log K = 7.3)
Luteolin				
Chrysin				
Rutin				
Naringin				
Genistein				
Biochanin A				
Kaempferol				
Naringenin				

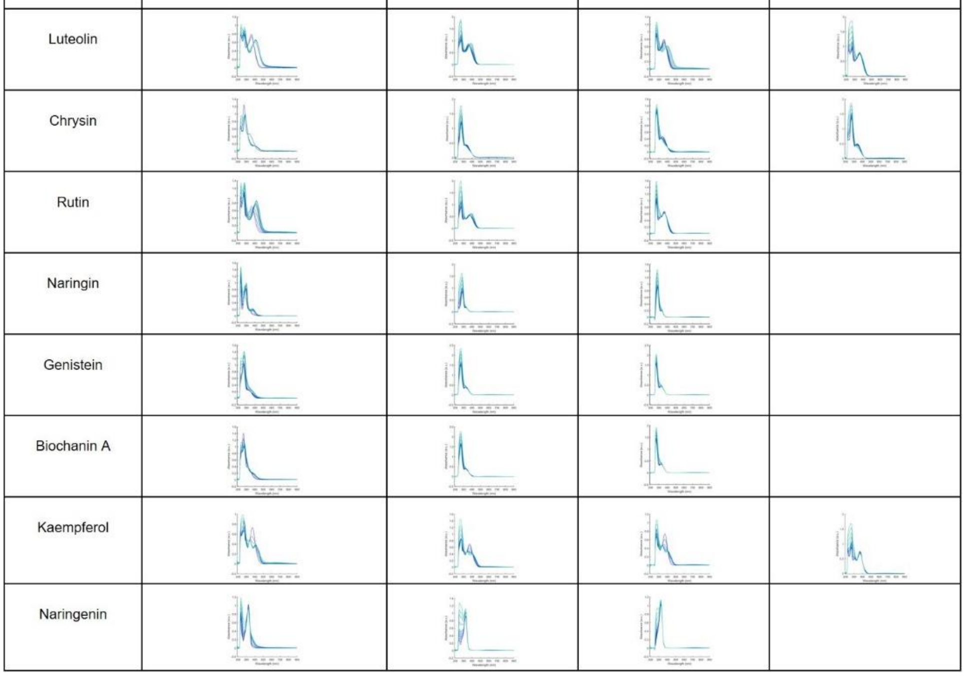

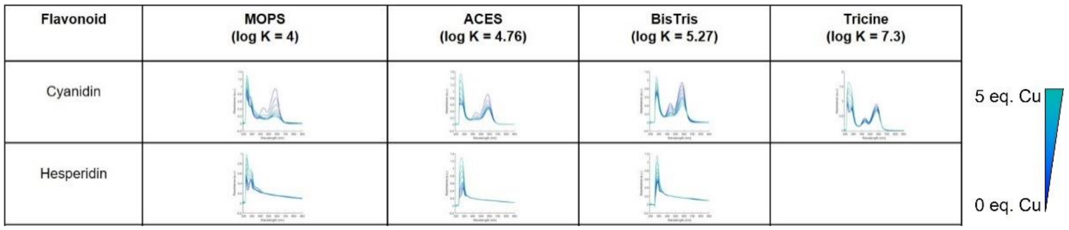

Electronic absorption spectra of flavonoid solutions titrated with $CuSO_4$ in buffers MOPS, ACES, BisTris, and Tricine (pH 7.4). Flavonoids were prepared at 50 µM and $CuSO_4$ was titrated into the solution from 0.2 to 5 molar equivalences.

SI Figure 2.

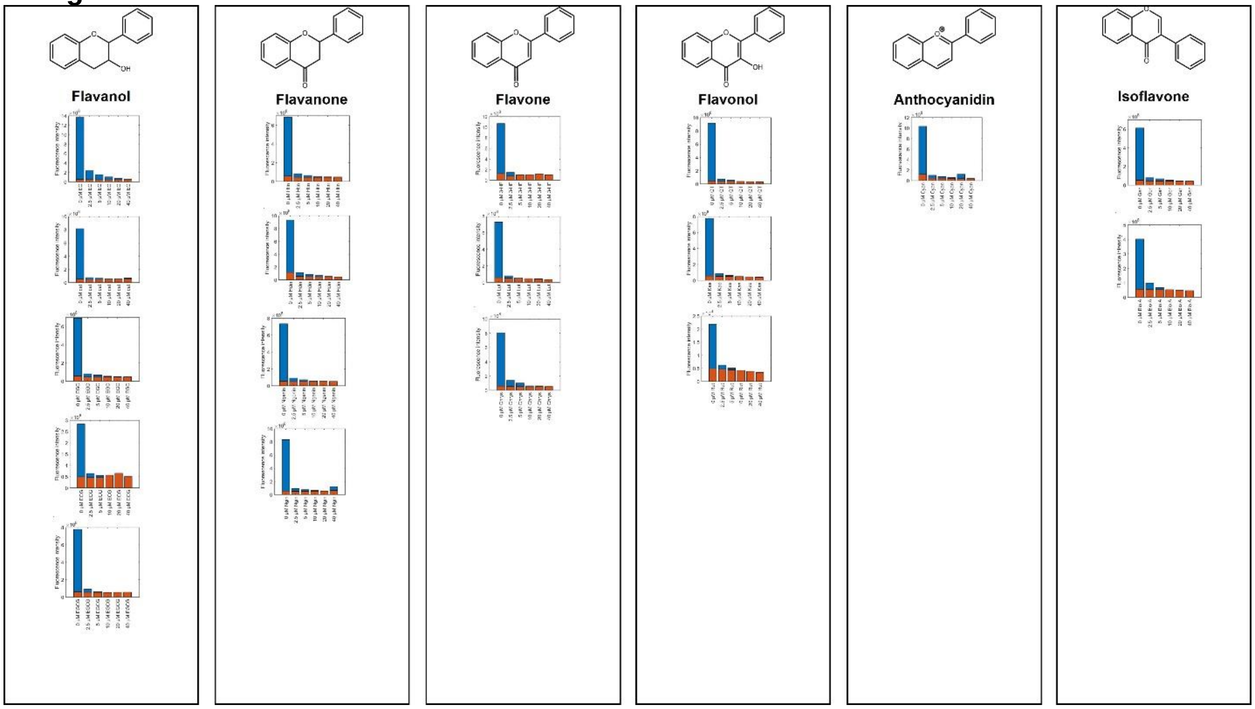

CCA fluorescence intensity measurements for determining the effects of flavonoids on the time-dependent generation of •OH. Flavonoid effects were compared to Cu(II) addition alone and with addition of flavonoid. 2.5 mM CCA and 50 µM ascorbic acid were used for all experiments. The calculated area under the curve over the course of 90 minutes was measured for flavonoid solution. Flavonoid effects are shown in the presence (blue) and absence (red) of Cu(II).

SI Figure 3.

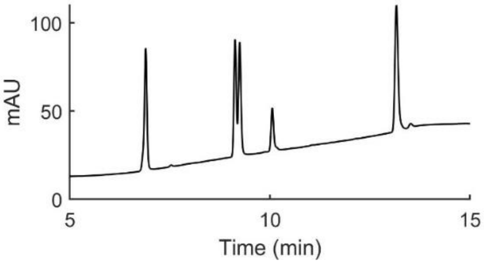

Elution time (min)	Flavonoid
6.896	EGCG
9.129	Luteolin
9.244	Quercetin
10.058	Kaempferol
13.160	3-hydroxyflavone

A mixture of 50 μM flavonoids was prepared in methanol. A gradient liquid chromatography experiment was performed ramping from 90:10 H_2O:acetonitrile to 10:90 H_2O:acetonitrile. The order of elution supports the relative hydrophobicities reported previously (SI Table 1).

SI Table 1. Reported log P values of Cu(II)-binding flavonoids

Flavonoids	Log P
EGCG	0.46(Arrest and Human 2020)
3-HF	4.17(Pogodaeva et al. 2012)
Luteolin	0.7(Quintieri et al. 2008)
QT	1.82(Rothwell, Day, and Morgan 2005)
Kaempferol	1.872(Sreelakshmi, Raj, and Abraham 2017)

SI Table 2. Strains of Saccharomyces cerevisiae used in this study

Strain	Genotype	Source
BY4741 WT	MATa, his3Δ1, leu2Δ0, met15Δ0, ura3Δ0	Horizon
BY4741 *ctr1Δ*	MATa, his3Δ1, leu2Δ0, met15Δ0, ura3Δ0, *ctr1Δ*	Horizon
BY4741 *ccc2Δ*	MATa, his3Δ1, leu2Δ0, met15Δ0, ura3Δ0, *ccc2Δ*	Horizon

SI Figure 4.

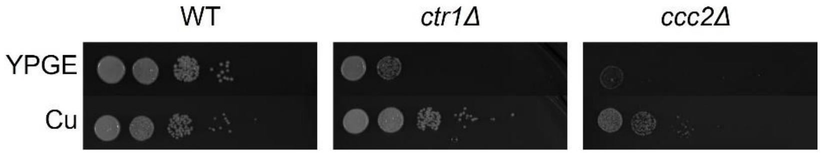

Serially diluted *S. Cerevisiae* strains were spotted on the noted YPGE plates and incubated at 37°C for 4 days before imaging. 10 μM $CuSO_4$ was supplemented onto YPGE agar plates. Copper treatment rescues growth of both *ctr1* and *ccc2* yeast knockout strains.

SI Table 3. p-values versus the vehicle control for the MTS assay and ICP-OES analysis

	MTS assay			ICP-OES analysis		
**Treatment**	**mean**	**SD**	**p-value**	**mean**	**SD**	**p-value**
Control	0.7474	0.0891		255.8	65.78	
EGCG	1.0251	0.1120	0.0104	159.8	54.75	0.1238
3-hydroxyflavone	0.8130	0.1008	0.3669	241.1	77.30	0.8139
Luteolin	0.6096	0.0735	0.0544	205.6	50.90	0.3543
Quercetin	0.8473	0.0604	0.1129	192.8	39.85	0.2290
Kaempferol	0.8490	0.0680	0.1199	192.6	28.78	0.2017
Cu Control	0.9208	0.0681		1.88×10^3	409.0	
EGCG + Cu	0.9270	0.0330	0.8753	1.86×10^3	617.7	0.9590
3-hydroxyflavone + Cu	0.1552	0.0148	5.83×10^{-7}	3.94×10^3	803.3	0.0166
Luteolin + Cu	0.7399	0.0650	0.0085	1.39×10^3	544.9	0.2824
Quercetin + Cu	0.9456	0.0600	0.6057	1.61×10^3	824.7	0.6396
Kaempferol + Cu	0.9947	0.1237	0.3362	1.49×10^3	789.9	0.4970

SI Figure 5.

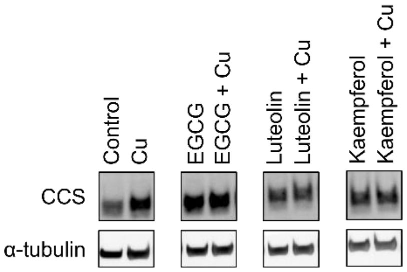

HepG2 cells were stimulated with 20 μM flavonoid with and without 50 μM CuSO4 and incubated for 24 hours. Cell lysates were collected and Western blot analysis was performed using antibodies specific for CCS with α-tubulin as a control.

References

Arrest, Cycle, and D U Human. 2020. "Lipophilized Epigallocatechin Gallate Derivative."

Pogodaeva, N. N., S. A. Medvedeva, B. G. Sukhov, and L. I. Larina. 2012. "Spectroscopic Study of the Reaction of a Natural Arabinogalactan Polysaccharide with 3-Hydroxyflavones in Aqueous Solutions." *Chemistry of Natural Compounds* 48 (5): 723–27. https://doi.org/10.1007/s10600-012-0368-0.

Quintieri, Luigi, Pietro Palatini, Alberto Nassi, Paolo Ruzza, and Maura Floreani. 2008. "Flavonoids Diosmetin and Luteolin Inhibit Midazolam Metabolism by Human Liver Microsomes and Recombinant CYP 3A4 and CYP3A5 Enzymes." *Biochemical Pharmacology* 75 (6): 1426–37. https://doi.org/10.1016/j.bcp.2007.11.012.

Rothwell, Joseph A., Andrea J. Day, and Michael R.A. Morgan. 2005. "Experimental Determination of Octanol-Water Partition Coefficients of Quercetin and Related Flavonoids." *Journal of Agricultural and Food Chemistry* 53 (11): 4355–60. https://doi.org/10.1021/jf0483669.

Sreelakshmi, Vasudevanpillai, Navya Raj, and Annie Abraham. 2017. "Evaluation of the Drug-like Properties of Kaempferol, Chrysophanol and Emodin and Their Interactions with EGFR Tyrosine Kinase - An in Silico Approach." *Natural Product Communications* 12 (6): 915–20. https://doi.org/10.1177/1934578x1701200621.
